# The Use of the General Animal-Based Measures Codified Terms in the Scientific Literature on Farm Animal Welfare

**DOI:** 10.3389/fvets.2021.634498

**Published:** 2021-06-04

**Authors:** Marta Brscic, Barbara Contiero, Luisa Magrin, Giorgia Riuzzi, Flaviana Gottardo

**Affiliations:** Department of Animal Medicine, Production and Health, University of Padova, Legnaro, Italy

**Keywords:** animal-based measure, animal welfare assessment, scientific literature, gap mapping, penetration level

## Abstract

**Background:** The approach to farm animal welfare evaluation has changed and animal-based measures (ABM), defined as the responses of an animal or effects on an animal, were introduced to assess animal welfare. Animal-based measures can be taken directly on the animal or indirectly and include the use of animal records. They can result from a specific event or be the cumulative outcome of many days, weeks, or months. The objective of the current study was to analyze the use of general ABM codified terms in the scientific literature, the presence of their definitions, and the gap mapping of their use across animal species, categories, years of publication, and geographical areas of the corresponding author's institution. The ultimate aim was to propose a common standard terminology to improve communication among stakeholders. In this study, data models were populated by collecting information coming from scientific papers extracted through a transparent and reproducible protocol using Web of Science^TM^ and filtering for the general ABM codified terms (or synonyms/equivalents). A total of 199 papers were retained, and their full texts were assessed. The frequency of general codified ABM terms was analyzed according to the classification factors listed in the objectives. These papers were prevalently European (159 documents), and the most represented species was cattle. Fifty percent of the papers did not provide a definition of the general ABM terms, and 54% cited other sources as reference for their definition. The results of the study showed a very low penetration of the general codified ABM term in the literature on farm animal welfare, with only 1.5% of the papers including the term ABM. This does not mean that specific ABM are not studied, but rather that these specific ABM are not defined as such under a common umbrella, and there is no consensus on the use of terminology, not even among scientists. Thus, we cannot expect the stakeholders to use a common language and a standardized terminology. The recognition and the inclusion of ABM in the lists of commonly accepted abbreviations of scientific journals could be a first step to harmonize the terminology in the scientific literature.

## Introduction

The first animal welfare assessment schemes were developed in the 1990s, and they were introduced within the organic farming assurance protocols (1, 2). At that time, these assessment schemes relied mainly on resources and management-based parameters to evaluate relations between environmental conditions and animal welfare (3). The framework of the animal welfare assessment was the evaluation of the farming conditions, and end-users drew conclusions on animal welfare based on the estimated relation between these conditions and the extent that these fulfilled the needs of the animals. These needs were represented by the Five Freedoms and their provisions: freedom from hunger and thirst—by ready access to fresh water and a diet to maintain full health and vigor; freedom from discomfort—by providing an appropriate environment including shelter and a comfortable resting area; freedom from pain, injury, or disease—by prevention or rapid diagnosis and treatment; freedom to express normal behavior—by providing sufficient space, proper facilities, and company of the animal's own kind; and freedom from fear and distress—by ensuring conditions and treatment which avoid mental suffering. In the meantime, the animal welfare scientists started developing and testing for application on farm measures based on direct observation of the animals and on animal records. Several research groups promoted indeed the need of an integrated approach in which both resource/management and animal-based measures (ABM) are necessary to assess animal welfare in a holistic way (4, 5). This is, in particular, due to the fact that animal welfare was recognized as a multidimensional concept that includes both the physical and mental state of the animal and that the Five Freedoms were considered as defining ideal states rather than standards for acceptable welfare (6). Therefore, researchers have probably used ABM as tools to assess animal needs long before they were conceptualized and classified under the umbrella of the general ABM codified term. The ABM were aimed at measuring the welfare status of the animal by assessing the outcomes. Indeed they can show the outcome of integrated resource and management factors in the experience of the animal itself (7) and can therefore be a more valid measure of welfare (8). In dairy cows, for example, the approach changed in such a way that the direct assessment of the animal, by measuring the time needed to lie down, was preferred over measuring size, softness, and other characteristics of the cubicles as it was more valid in evaluating the real welfare state of the animal (9). The development of several valid ABM and their classification under a common terminology were the main achievements of the Welfare Quality project. After Welfare Quality, other research projects focusing on ABM were financed by the European Union (EU) within the 7^th^ and Horizon 2020 framework programs. Most projects considered assessment of animal welfare on farm, either directly or retrospectively at slaughter (e.g., AWIN, AssureWel, PROHEALTH ClearFarm, different COST Actions, etc.). The European Food and Safety Authority (EFSA) considered the use of ABM in the assessment of animal welfare so relevant that it commissioned a statement in order to establish a common framework for future scientific opinions and to clarify some common issues on the terminology and integration of concepts (6). According to this statement, ABM are defined as the response of an animal or an effect on an animal used to assess its welfare. They can be taken directly on the animal or indirectly and include the use of animal records. They can result from a specific event, e.g., an injury, or be the cumulative outcome of many days, weeks, or months, e.g., body condition. Further pilot projects were commissioned by EFSA to point out the need for context-specific ABM as, for example, the case of small mountain dairy farms where ABM developed through the Welfare Quality project were not directly applicable to this context (10). Animal-based measures were also included in some EU animal welfare legislative acts, commentary documents from NGOs, and assurance schemes (e.g., those developed by AssureWel, RSPCA, Biobord, RedTractors).

The rationale behind this study comes from the evidence of the wide use of ABM by public institutions and governments, in dedicated EU projects, in a range of quality assurance schemes, in EU legislative acts for the protection of animals (11), and by animal protection NGOs and from the increasing awareness of the need for scientific validity of these measures. Therefore, the objective of the current study was to analyze the use of the general ABM codified terms in the scientific literature on farm animal welfare since their first conceptualization, along with the presence of their definitions and the gap mapping of their use across different animal species, categories (e.g., within cattle dairy, beef, calf), years of publication, and geographical areas of the institution of the corresponding author. The ultimate aim was to propose a common standard terminology to improve communication and facilitate the connections among stakeholders.

## Materials and Methods

### Scientific Literature Search

A literature search protocol was set up by using Web of Science—© 2020 Clarivate Analytics to identify peer-reviewed papers that were written in English and that covered the topic of farm animal welfare. The search was performed in June 2020. The basic inclusion criteria for the selection of the peer-reviewed papers were as follows:

- Timespan = 1990–2019
- Search language = English
- Search topics = title, abstract, author keywords, keywords plus

**Figure 1:**
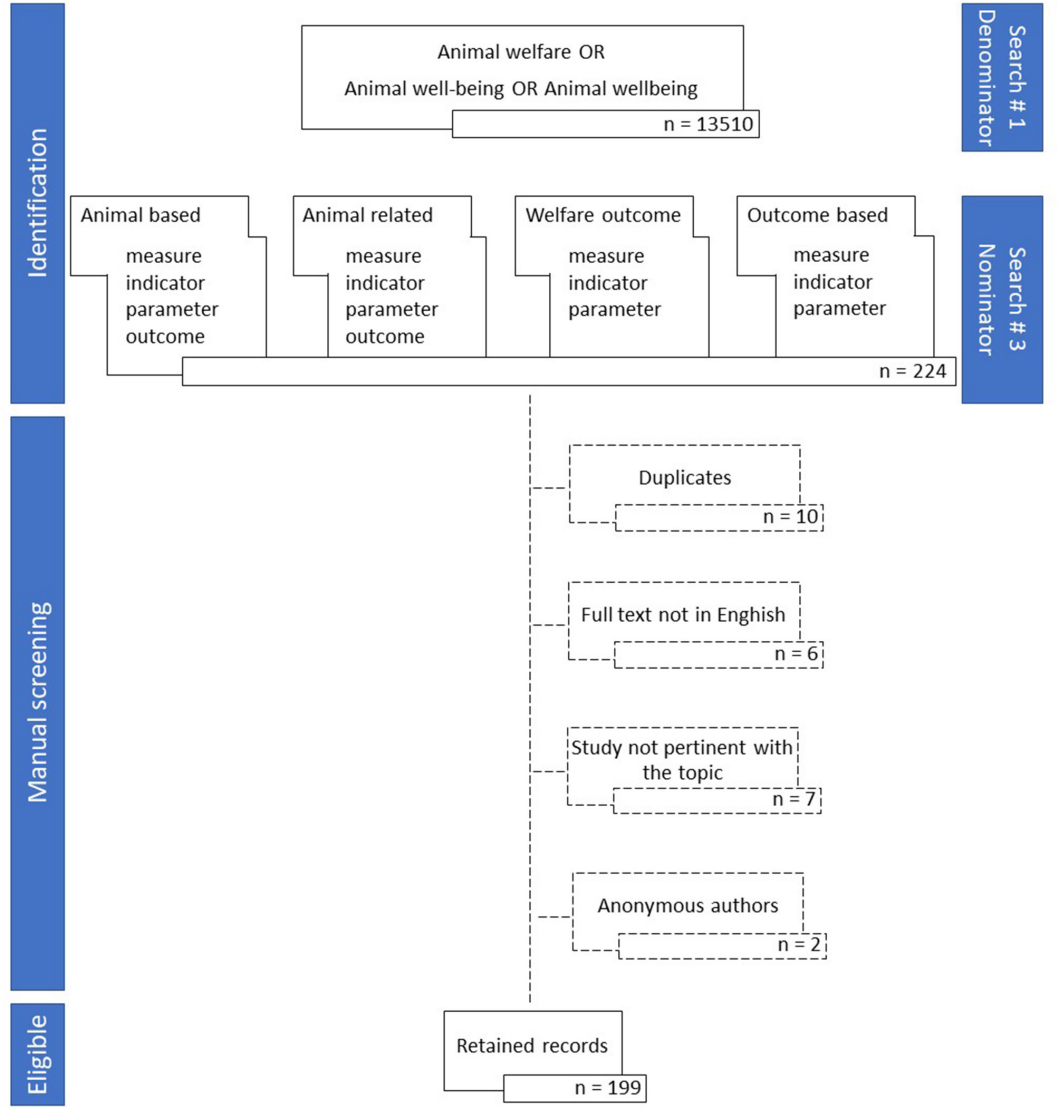
Flow chart graphically representing the search protocol. The dashed line represents the papers excluded at manual screening.

The flow chart shown in Figure 1 describes the search protocol that was developed through the following steps:

- selection of papers containing “animal welfare” OR “animal well-being” OR “animal wellbeing” (first search string)
- exclusion of papers dealing with “animal welfare”, “animal well-being”, or “animal wellbeing” in human-related studies. The exclusion criteria adopted were based on a search string that excluded “wild animal,” “marine mammal”, “pet animal”, “laboratory animal”, “companion animal”, “zoo animal”, “dog”, “cat”, “mouse”, “mice”, “rat”, or “rodent” (second search string)
- selection of papers containing the general codified ABM term or all the potential synonyms/equivalents of ABM (animal related, welfare outcome, and outcome based) considered by Gottardo et al. (11) within papers containing “animal welfare,” “animal well-being,” or “animal wellbeing.” The search string applied was (“animal welfare” OR “animal well-being” OR “animal wellbeing) AND (((“animal based” OR animal-based) NEAR/3 measure^*^) OR ((“animal based” OR animal-based) NEAR/3 indicator^*^) OR ((“animal based” OR animal-based) NEAR/3 outcome^*^) OR ((“animal based” OR animal-based) NEAR/3 parameter^*^) OR ((“animal related” OR animal-related) NEAR/3 measure^*^) OR ((“animal related” OR animal-related) NEAR/3 indicator^*^) OR ((“animal related” OR animal-related) NEAR/3 outcome^*^) OR ((“animal related” OR animal-related) NEAR/3 parameter^*^) OR ((“welfare outcome” OR “outcome based” OR outcome-based^*^) NEAR/3 measure^*^) OR ((“welfare outcome” OR “outcome based” OR outcome-based^*^) NEAR/3 indicator^*^) OR ((“welfare outcome” OR “outcome based” OR outcome-based^*^) NEAR/3 parameter^*^)) (third search string). NEAR/*x* = finds records where the terms joined by the operator are within a specified number of words from each other. *x* is the maximum number of words that separates the terms [i.e., = (“animal based” NEAR/3 measure^*^) finds all the records where in a given sentence they are separated by no more than three words as in the case of “animal based welfare assessment measures”].

In line with the aim of this study, which is to standardize the terminology, we refer to:

- “animal welfare” for the broad first-level set of terms “animal welfare/animal well-being/animal wellbeing”
- “general ABM root term” for the second-level set of terms “Animal based/animal-based (AB), Animal related/animal-related (AR), Welfare Outcome (WO), and Outcome based/outcome-based (OB)”
- “general ABM ending term” for the third-level set of terms “Measure/Indicator/Parameter/Outcome”
- “general ABM codified term” for the combination of the “general ABM root term” and “general ABM ending term.” From here onwards, we will use this categorization.

**Figure 2:**
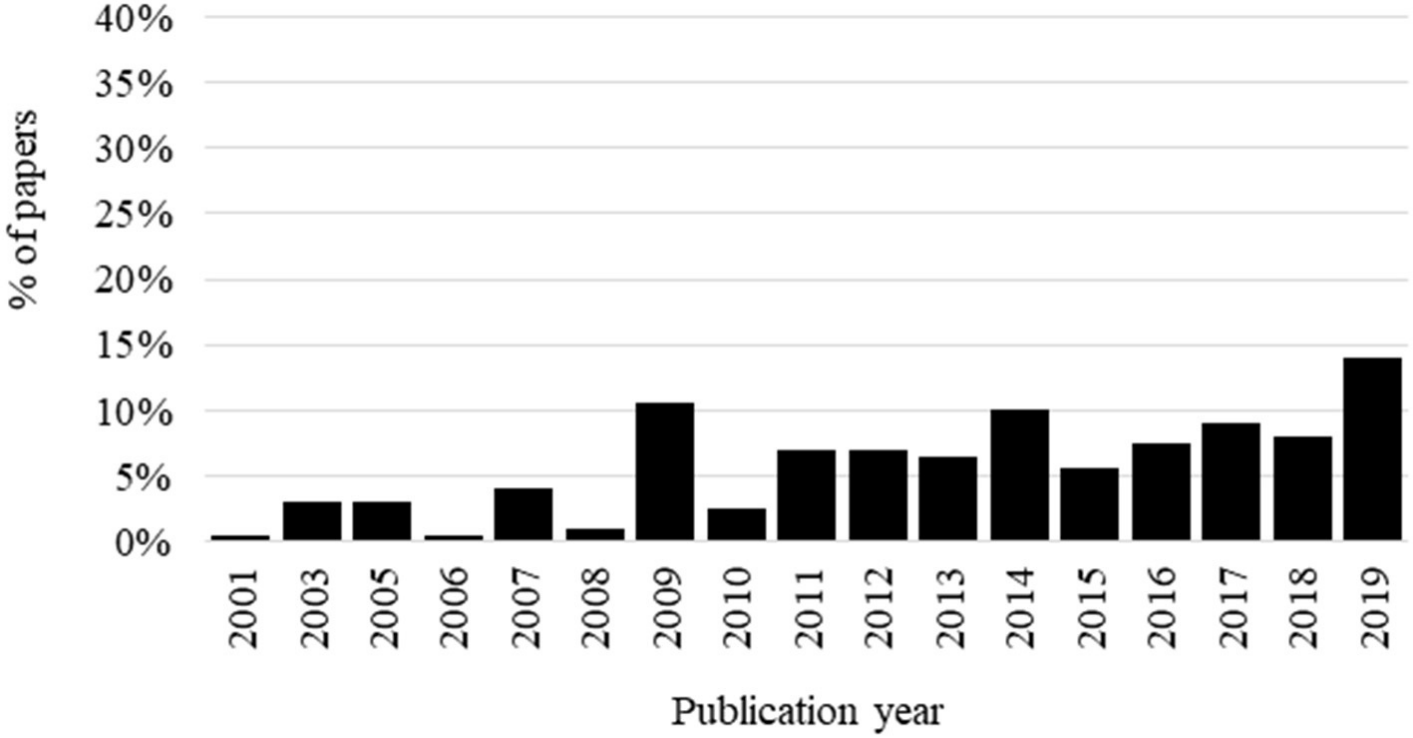
Percentage of papers including a general animal-based measures codified term according to the publication year.

The retained records were then submitted to a manual screening that had different purposes. As graphically presented in Figure 1, the first manual screening was performed to clean the dataset, thus, to eliminate the records whose full text was not in English, duplicates, anonymous authors, and/or that were not pertinent to the topic. Once the eligible documents were retained, the full text was analyzed to classify each paper according to animal species and production category, type of study, scenario, and application in organic farming. The levels and the descriptions of the classification factors are reported in Table 1. The further analysis of the full text included (1) the identification of presence of one or more ABM general term (yes/no/not reported for each of the codified terms reported in Figure 1), (2) presence of a definition of the ABM general term provided by the authors (yes/no) or definition referring to a citation (yes/no), (3) in case the definition was referring to a citation, the reference was copied and pasted, and (4) presence of specific ABM in the full text, figures, or tables (yes/no) regardless of the specific ABM name, form, or unit [e.g., a study dealing with lameness was reported as including a specific ABM (yes), although it could include mild lameness, severe lameness, lameness prevalence, lameness scoring, percentages of lame animals within each score, and/or different scoring systems]. Reporting the specific ABM was not within the scope of this paper. The full texts were assessed by four assessors trained in a standard procedure to screen the papers and to fill in a shared Excel document with drop-down lists in order to have a common systematic criterion of evaluation and data collection. Each assessor evaluated individually an equal number of records. In case of doubts, the evaluators discussed among each other to make a final decision. The full list of retained documents is provided as Supplementary Material.

**Table 1:** Factors used to classify the papers, the different levels, and the explanation of how each paper was classified.

**Factor**	**Levels**	**Explanation**
Animal species	Cattle Equine Fur animals Goat Poultry Rabbit Sheep Swine Other General	Each paper was classified according to the main farm animal species it dealt with, and each animal species was further classified according to the animal category (e.g., cattle was further subdivided in dairy, beef, and calf; swine was further subdivided in fattening pig, sow, piglet/other). A paper was classified as other if it dealt with other minor animal species or as general if it was of a general wide approach and not involving given animal species. A paper dealing with more than one species was classified in more than one class
Type of study	Methodological Research Assessment Other	Each paper was classified as methodological if it described a method applied or the development of a methodology (e.g., validation), as a research if it was an original applicative study with data produced by the research, as an assessment if it described an animal welfare assessment or its application, and as other if it did not fall in any of these classifications. A single paper was classified in more than one class if it considered more than one of the aspects listed
Scenario	On farm At slaughter During transport Not reported	Each paper was classified according to its scenario of application or with the scenario it dealt with: on farm, at slaughter, and/or during transport. A single paper was classified in more than one class if it considered more than one scenario
Organic farming	Yes No Not reported	Each paper was classified as dealing with organic farming (yes) if it included the application in organic farms (according to organic principles) or of it dealt with comparisons of conventional (no) vs. organic production systems (yes) and as not organic (no) if the application was on conventional farms and as not reported if it was not specified in the full text

Data were submitted to descriptive statistics by using Excel/STAT and considering publication year, animal species and category, scenario, and application in organic farming as classification factors. This approach was adopted also in order to carry out the gap mapping of the distribution of the ABM in the scientific literature across the different classification factors.

The gap mapping was carried out over the following subsequent steps: (1) identification of the problem area in the use of terminology related to the general ABM term, (2) definition of the goal for which at least animal welfare scientists could use a common terminology worldwide, (3) determination of the current use of the terminology within literature on animal welfare, (4) determination of a potential desired homogeneous use, and (5) identification of the gaps between the two uses.

An indication of how the use of terminology related to the general ABM term penetrated the scientific literature dealing with animal welfare was obtained by calculating the following two ratios:

- the ratio between papers with the general ABM term (nominator) and the total number of papers on animal welfare (denominator) and
- the ratio between total citations and the number of papers for each general term: average number of citations per paper.

## Results and Discussion

The results of the literature search strings are reported in the flow chart in Figure 1, along with the number of records per each search combination. The final outcome of the set-up procedure described in section “Materials and Methods” identified 199 scientific papers that were retained for data collection and calculation of the penetration indexes. The ratio between papers with the general ABM term over the papers on animal welfare is 1.5%, and it is likely to indicate an overall low level of penetration of the ABM general term in the scientific literature on animal welfare. This result might not necessarily indicate a low use of specific ABM (e.g., body condition score, mortality, cleanliness, lameness) but rather that animal welfare scientists do not classify the specific ABM as such using a common terminology. Differences in the penetration of the use of the general ABM terms in the scientific literature were observed when analyzing the distribution of the papers in relation to the geographical area of the institution of the corresponding author. The majority of papers were from Europe (159 documents, 80%) followed by America (27 documents, 14%), Oceania (nine documents, 4%), and Asia (four documents, 2%). None of the documents, including the general ABM terms, were attributed to correspondence of African institutions. This overview is likely to reflect the fact that the European scientific community is more used to network for applications to project funding from EU, promoting a more homogeneous use of technical terminology. Once the general ABM term was conceptualized (5), several research groups probably started using it as an umbrella term. Indeed, as reported in Figure 2, the papers including the general ABM terms were published as of 2001, and the first paper referred to a European COST Action as funding source. The percentage of selected papers according to the publication year is reported in Figure 2. A peak was detected in 2009, when the Welfare Quality project ended with the publication of the welfare assessment protocols and most of the research groups active in the project published their papers. This could have determined an increasing trend in the subsequent years, with a new peak observed in 2019. The documents were published on 54 different scientific journals, making evident a scattered distribution of the general codified term also in journals that are not specialized in the animal welfare field. Eight journals published five or more papers containing the general ABM codified term (Table 2). Animal Welfare of the University Federation for Animal Welfare, a highly specialized journal, was the most represented in this list. The other journals have a more general approach, and they publish cross-cutting topics on animal science.

**Table 2:** Number and percentage of papers including a general animal-based measures codified term published in the journals with more than five papers.

**Journal**	**Number**	**Percentage (%)**
*Animal Welfare*	54	27
*Animals*	16	8
*Animal*	15	7
*Journal of Dairy Science*	13	6
*Italian Journal of Animal Science*	11	5
*Journal of Animal Science*	7	4
*Poultry Science*	7	4

The retained peer-reviewed papers were classified as original research papers (63 documents, 32%), methodological studies (63 documents, 32%), assessments (57 documents, 29%), and/or as other if they could not be characterized within any of the above-mentioned macro groups (43 documents, 22%). We expected a more homogenous use of terminology and a more frequent appeal to the general ABM terms in methodological and in assessments studies compared to original research papers. This expectation was not met by our results, and the frequency distribution did not show a high prevalence of specific types of papers, probably indicating that scientists are still exploring all these three aspects, the assessment scheme application (12, 13), research on ABM (14–16), or development of methodological aspects linked to ABM (17–19). However, listing a paper in only one of these classification groups was sometimes difficult.

**Figure 3:**
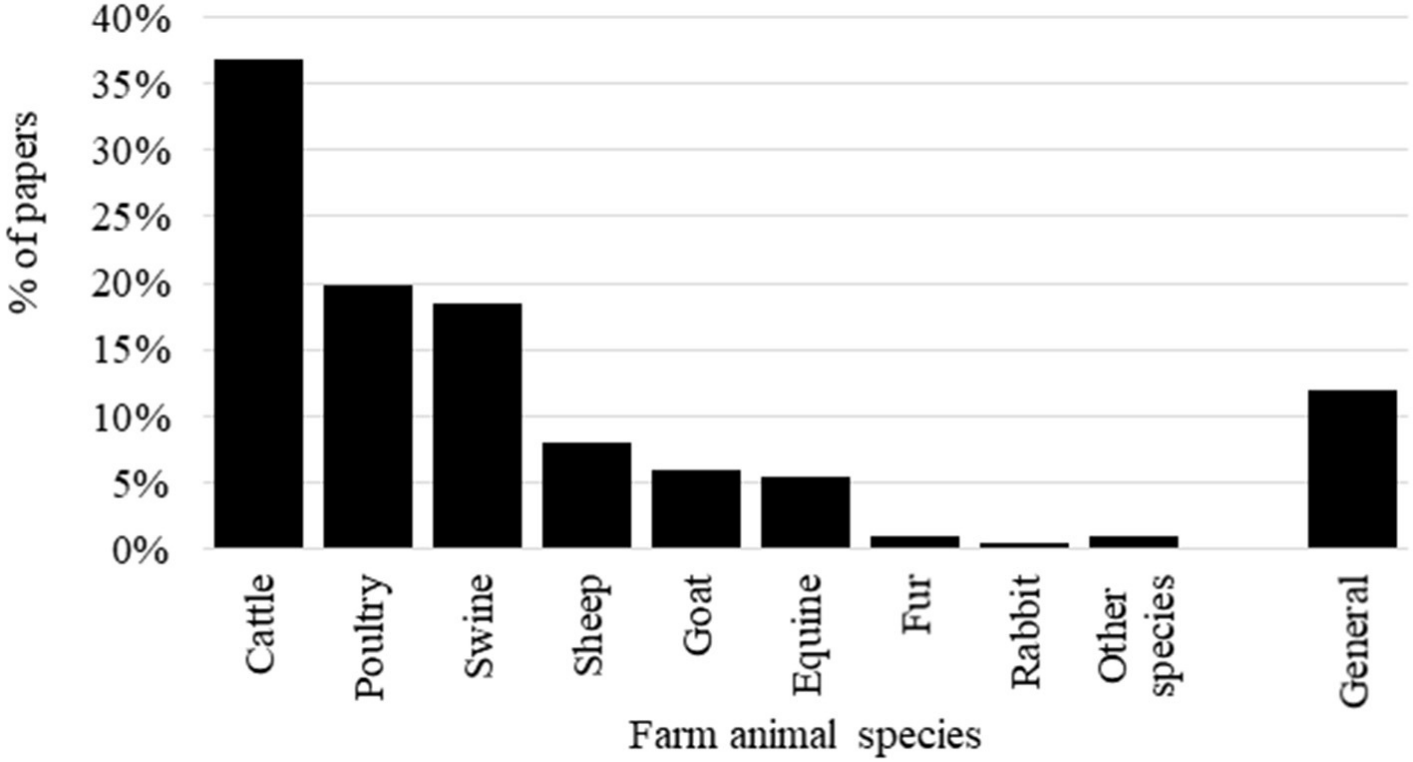
Percentage of papers including a general animal-based measures codified term according to the animal species.

**Figure 4:**
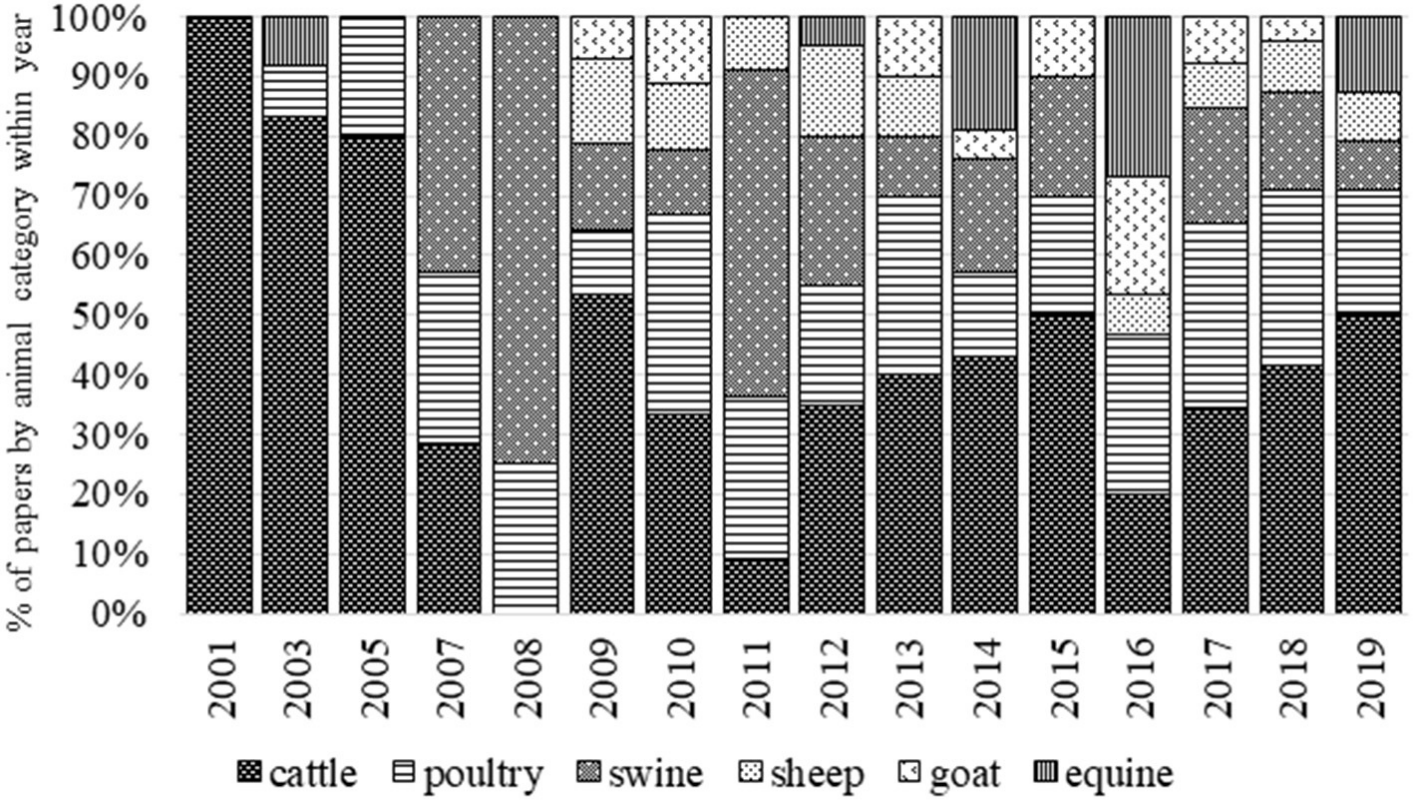
Frequency distribution of papers including a general animal-based measures codified term according to the animal species within the year of publication.

The number and percentage of papers per animal species are reported in Figure 3. More than one species were included in nine papers, while 24 papers were of a general methodological approach (without any specific species analyzed). Cattle, poultry, and swine are the most represented species in the literature including a general ABM term. The first approach to the definition of the general ABM term was addressed in cattle in 2001 (Figure 4). Thus, it is likely that this terminology was largely used in literature on cattle, therefore causing this greater number of documents. Among cattle, the largest number of papers with ABM regarded dairy cows (67%), beef (11%), and calf (9%). Papers dealing with calves were on dairy calves, not veal. These results likely suggest that, in the past, cattle is the animal category that, in fact, has shown more need for ABM than other categories for the wide options of their housing and rearing systems (e.g., from pasture-based to indoor loose cubicle housing) in which the same evaluation method based on resource- and management-based measures was not directly applicable (5, 20). Indeed the literature on dairy cattle deals with the development of assessment protocols in small-scale mountain farms (21, 22), pasture-based systems (23, 24), or specific problems in indoor farms, e.g., lameness, mastitis, etc. (25–27). The second most represented group of species is poultry. Among poultry, broiler chicken (61%) and lying hens (39%) are involved in the vast majority of the papers, whereas duck, goose, and turkey are marginal (3% of the overall papers on poultry). This result does not mean again that specific ABM are not used in studies on these categories of animals, but only that they are not codified as such. Documents on poultry deal with studies on different husbandry systems including free-range and organic scenarios (28–30). As shown in Figure 4, papers on sheep, goat, and equine emerged in the timeframe that is subsequent to the outset of the AWIN project that aimed at developing assessment schemes for these species. As regards swine, the scientific literature focused mainly on fattening pigs and piglets (70%) rather than on sows and gilts. We expected a higher prevalence of papers on fattening pigs, which is likely due to the awareness of public opinion about tail docking and castration that promoted studies addressing the development of alternative production practices (31). However, the published papers including the general ABM term covered topics that were mainly on assessment scheme applications on farm, during transport, and at slaughter and the testing of intra- and inter-observer reliability (18, 32, 33). It is likely that the scientific studies on mutilations and pain management do not refer to the general ABM terms.

The number of papers with different scenarios of application is reported in Table 3. The large majority of the papers regarded on-farm studies. Among the 11 studies applied on more than one scenario, eight studies regarded activities on farm and at the slaughter, and three studies were applied during transport and at the slaughter. The frequency distribution of the scenarios is likely reflecting the fact that assessment schemes implying the use of ABM aim at evaluating the level of animal welfare on farm, regardless of the site of its application. Specific ABM developed to be used at slaughter may aim either at a retrospective evaluation of the welfare on farm (9, 34, 35) or during transport (36) or at assessing the welfare at the time of slaughter and related operations (37).

**Table 3:** Number and percentage of papers including a general animal-based measures codified term according to the different scenarios.

**Scenario**	**Number**	**Percentage (%)**
On farm	154	77
At slaughter	12	6
During transport	2	1
More than one scenario	11	6
Not reported	20	10

Organic farming systems are not significantly represented among documents retained in this study (four documents, 2%), whereas both organic and conventional systems were present in 19 documents (9.4%). The adoption of the organic system of production was not reported in almost 60% of the papers. This result does not meet our assumption that papers describing studies in organic production systems include the general ABM term since the first animal welfare assessment schemes were introduced within the assurances of organic farming. This might be due to the evidence that organic assurance schemes relied mainly on resources and management-based parameters as required, for example, by EU legislation (38).

Among the different general ABM root terms searched in the retained documents, AB was the most frequently used, followed by WO and OB, whereas AR was the less used one (Table 4). The most frequently used general ABM ending term was measure(s) followed by indicator(s), parameter(s), and outcome(s). Outcome was the less used term, which is likely due to the lower number of combinations with the general ABM root term for semantic reasons (e.g., WO and OB outcome). The matrix of the general ABM codified term made of combinations of roots (AB, AR, WO, and OB) and endings [measure(s), indicator(s), outcome(s), and parameter(s)] is reported in Table 4. The most frequent general ABM codified term used in combination was Animal based/animal-based measure(s), and this could be expected considering that the Welfare Quality project opted for this terminology in its outputs. On the other hand, Animal based/animal-based indicator(s) was the terminology preferentially used in the AWIN project. Cross-use of the terminology is not rare; indeed two or three general ABM codified terms were used in 38 (19%) and 3 (1%) papers, respectively, whereas still a single general codified term was used in the majority of papers (154 documents, 77%).

**Table 4:** Number of documents and percentage (in brackets), total citations, and average number of citations per paper according to the general animal-based measures (ABM) root, ending and codified terms.

			**General ABM root term^b^**				
		**Papers in which the general ABM ending term is used^a^**	**Animal based/animal-based (AB)**	**Animal related/animal-related (AR)**	**Welfare outcome (WO)**	**Outcome based/outcome-based (OB)**	**Number of papers in which more than one term was used**
Papers in which the general ABM root term is used^a^			172 (87%)	16 (8%)	25 (13%)	24 (12%)	
General ABM ending term	Measure(s)	139 (70%)	122 (71%)	3 (19%)	18 (72%)	21 (87%)	23
	Parameter(s)	48 (24%)	43 (25%)	10 (62%)	0 (0%)	0 (0%)	4
	Indicator(s)	74 (37%)	67 (39%)	6 (38%)	8 (32%)	3 (13%)	10
	Outcome(s)	9 (5%)	9 (6%)	0 (0%)			0
Total citations (TC)		2,682	417	382	521		
TC/number of papers		15.6	26.1	15.3	21.7		

a *Overall percentage expressed on the total number of 199 retained documents*.

b *Percentage expressed on 172 (AB), 16 (AR), 25 (WO), and 24 documents (OB), respectively*.

Further indicators of the level of penetration of the general ABM terms in the scientific literature, which reflect the interest by the scientific community toward this topic, could be the total citations and the average number of citations per paper. Total citation is the number of times that a paper was cited in other scientific publications from its publication year until 2019 when the current literature search was carried out. The total citations collected by the scientific corpus of the 199 retained papers was 2,983. The citation indicators according to the general ABM root terms are reported in Table 4. The total citations were greater for AB, although the average citations per paper were greater for AR. Four documents had more than 100 citations (5, 39–41). Fifty percent of the papers did not provide a definition of the general ABM terms, and 54% cited one or more other sources as reference for the definition of general ABM terms. The most cited papers as reference for the definition with more than five citations among the literature corpus used in this paper are reported in Table 5. The mostly cited document is Welfare Quality (9), and this could be expected considering the wide use of the general ABM term compared to the other potential synonyms/equivalents. Surprisingly, 67 papers (33.3%) did not provide any explicit definition nor references to other sources.

**Table 5:** List of most cited papers (more than five times) as reference for the definition of the general codified animal-based measures term and number of documents in which they are cited in the corpus of the 199 retained papers.

	**Number of documents**
Welfare Quality® (9)	22
Whey et al. (39)	15
EFSA (6)	14
Main et al. (7)	8
Main et al. (42)	8
Johnsen et al. (43)	7
Webster et al. (44)	7
Blokhuis et al. (5)	6
Capdeville and Veissier (3)	6
Botreau et al. (45)	5
Keeling and Veissier (8)	5

In conclusion, the results of this study showed that the general ABM terms are used in a very limited fraction of the literature on animal welfare. In the scenario of the 199 papers including a general ABM codified term, the Welfare Quality project had the greatest impact; thus, the considered terminology and the species and categories of its application were the most represented. Fur animals, rabbits, and other niche farm animals were poorly represented in the retained documents of this literature corpus which could be expected by the limited number of farms and the localized productions. A different reason could support the fact that fish were almost absent, although there is a large number of farmed species and ABM are under development for aquatic organism. By looking at the source of the retained documents (journal of publication), it seems that the general codified ABM term is being used by experts involved in animal welfare studies, but it has also permeated scientists involved in animal production and other related topics where it could be expected that a common use of single specific measures is adopted, such as body condition, growth performance, cleanliness, and somatic cell count.

The implications of this study are linked to the fact that there is a huge amount of literature on animal welfare/well-being and a large body of literature that includes specific ABM (e.g., lameness, lesions, body condition, somatic cell count, mortality, etc.), but these specific ABM are not defined as such under a common umbrella, and there is no consensus on the use of terminology, not even among scientists. Thus, we could not expect stakeholders to use a common language and terminology. Going beyond the general terms, we expect that it could be even more difficult to have common names of specific ABM, which makes it even harder to define, standardize, or assess them for reliability, repeatability, reproducibility, robustness, feasibility, accuracy, sensitivity, specificity, and/or validity. In order to achieve the use of a common standard terminology in the future, some of the different possible ways forward could be their use by authorities in animal welfare legislations, by scientific societies dealing with animal behavior and welfare and animal sciences in general, and by NGOs, companies, and institutions involved in the development and application of quality assurance schemes. Moreover, the recognition and the inclusion of ABM in the lists of commonly accepted abbreviations of the scientific journals could be a first step to harmonize the terminology in the scientific literature.

## Author Contributions

All the authors contributed to data curation. FG, MB, and BC contributed to the conceptualization and writing of the original draft. BC performed the formal analysis. LM and GR contributed to the methodology. All authors contributed to the article and approved the submitted version.

## Conflict of Interest

The authors declare that the research was conducted in the absence of any commercial or financial relationships that could be construed as a potential conflict of interest.
