# Supplementary material for: The Use of the General Animal-Based Measures Codified Terms in the Scientific Literature on Farm Animal Welfare
Source: Front Vet Sci. 2021 Jun 4;8:634498. doi: 10.3389/fvets.2021.634498 (PMC8212950; doi:10.3389/fvets.2021.634498)
Supplement: Supplementary file 1 [file Table_1.DOC]

Aerts S, Lips D, Spencer S, Decuypere E, De Tavernier J. A new framework for the assessment of animal welfare: Integrating existing knowledge from a practical ethics perspective. *J Agric Environ Ethics* (2006) **19**:67–76. doi:10.1007/s10806-005-4376-y

Alban L, Petersen JV, Busch ME. A comparison between lesions found during meat inspection of finishing pigs raised under organic/free-range conditions and conventional, indoor conditions. *Porc Heal Manag* (2015) **1**:4.

Andreasen SN, Sandøe P, Forkman B. Can animal-based welfare assessment be simplified? A comparison of the welfare quality® protocol for dairy cattle and the simpler and less time-consuming protocol developed by the Danish Cattle Federation. *Anim Welf* (2014) **23**:81–94. doi:10.7120/09627286.23.1.081

Anzuino K, Bell NJ, Bazeley KJ, Nicol CJ. Assessment of welfare on 24 commercial UK dairy goat farms based on direct observations. *Vet Rec* (2010) **167**:774–780. doi:10.1136/vr.c5892

Armbrecht L, Lambertz C, Albers D, Gauly M. Assessment of welfare indicators in dairy farms offering pasture at differing levels. *Animal* (2019) **13**:2336–2347. doi:10.1017/S1751731119000570

Averós X, Aparicio MA, Ferrari P, Guy JH, Hubbard C, Schmid O, Ilieski V, Spoolder HAM. The effect of steps to promote higher levels of farm animal welfare across the EU. societal versus animal scientists’ perceptions of animal welfare. *Animals* (2013) **3**:786–807. doi:10.3390/ani3030786

Barnett JL, Hemsworth PH. Welfare monitoring schemes: Using research to safeguard welfare of animals on the farm. *J Appl Anim Welf Sci* (2009) **12**:114–131. doi:10.1080/10888700902719856

Barry J, Kennedy E, Sayers R, De Boer IJM, Bokkers EAM. Development of a welfare assessment protocol for dairy calves from birth through to weaning. *Anim Welf* (2019) **28**:331–344. doi:10.7120/09627286.28.3.331

Bassler AW, Arnould C, Butterworth A, Colin L, De Jong IC, Ferrante V, Ferrari P, Haslam S, Wemelsfelder F, Blokhuis HJ. Potential risk factors associated with contact dermatitis, lameness, negative emotional state, and fear of humans in broiler chicken flocks. *Poult Sci* (2013) **92**:2811–2826. doi:10.3382/ps.2013-03208

Battini M, Barbieri S, Fioni L, Mattiello S. Feasibility and validity of animal-based indicators for on-farm welfare assessment of thermal stress in dairy goats. *Int J Biometeorol* (2016) **60**:289–296. doi:10.1007/s00484-015-1025-7

Battini M, Stilwell G, Vieira A, Barbieri S, Canali E, Mattiello S. On-farm welfare assessment protocol for adult dairy goats in intensive production systems. *Animals* (2015) **5**:934–950. doi:10.3390/ani5040393

Battini M, Vieira A, Barbieri S, Ajuda I, Stilwell G, Mattiello S. Invited review: Animal-based indicators for on-farm welfare assessment for dairy goats. *J Dairy Sci* (2014) **97**:6625–6648. doi:10.3168/jds.2013-7493

Beggs DS, Jongman EC, Hemsworth PH, Fisher AD. The effects of herd size on the welfare of dairy cows in a pasture-based system using animal- and resource-based indicators. *J Dairy Sci* (2019) **102**:3406–3420. doi:10.3168/jds.2018-14850

Berckmans D. General introduction to precision livestock farming. *Anim Front* (2017) **7**:6–11. doi:10.2527/af.2017.0102

Bergman P, Munsterhjelm C, Virtala AM, Peltoniemi O, Valros A, Heinonen M. Structural characterization of piglet producing farms and their sow removal patterns in Finland. *Porc Heal Manag* (2019) **5**:12. doi:10.1186/s40813-019-0119-8

Bergmann S, Louton H, Westermaier C, Wilutzky K, Bender A, Bachmeier J, Erhard M, Rauch E. Field trial on animal-based measures for animal welfare in slow growing broilers reared under an alternative concept suitable for the German market. *Berl Munch Tierarztl Wochenschr* (2016) **12**:453–461. doi:10.2376/0005-9366-16035

Blatchford RA, Fulton RM, Mench JA. The utilization of the Welfare Quality® assessment for determining laying hen condition across three housing systems. *Poult Sci* (2016) **95**:154–163. doi:10.3382/ps/pev227

Blatchford RA. Animal behavior and well-being symposium: Poultry welfare assessments: Current use and limitations. *J Anim Sci* (2017) **95**:1382–1387. doi:10.2527/jas2016.0957

Blokhuis HJ, Veissier I, Miele M, Jones B. The welfare quality® project and beyond: Safeguarding farm animal well-being. *Acta Agric Scand A Anim Sci* (2010) **60**:129–140. doi:10.1080/09064702.2010.523480

Bozzo G, Barrasso R, Grimaldi CA, Tantillo G, Roma R. Consumer attitudes towards animal welfare and their willingness to pay. *Vet Ital* (2019) **55**:289–297. doi:10.12834/VetIt.1823.9669.2

Bracke MBM. Animal-based parameters are no panacea for on-farm monitoring of animal welfare. *Anim Welf* (2007) **16**:229–231.

Bright A, Brass D, Clachan J, Drake KA, Joret AD. Canopy cover is correlated with reduced injurious feather pecking in commercial flocks of free-range laying hens. *Anim Welf* (2011) **20**:329–338.

Broom D. Animal welfare: concepts, study methods and indicators. *Rev Colomb Ciencias Pecu* (2011) **24**:306–321.

Broom DM. Cow welfare and herd size: Towards a sustainable dairy industry. *Cattle Pract* (2014) **21**:169–173.

Browman HI, Cooke SJ, Cowx IG, Derbyshire SWG, Kasumyan A, Key B, Rose JD, Schwab A, Skiftesvik AB, Don Stevens E, et al. Welfare of aquatic animals: Where things are, where they are going, and what it means for research, aquaculture, recreational angling, and commercial fishing. *ICES J Mar Sci* (2019) **76**:82–92. doi:10.1093/icesjms/fsy067

Brscic M, Gottardo F, Tessitore E, Guzzo L, Ricci R, Cozzi G. Assessment of welfare of finishing beef cattle kept on different types of floor after short-or long-term housing. *Animal* (2014) **9**:1053–1058. doi:10.1017/S1751731115000245

Buijs S, Ampe B, Tuyttens FAM. Sensitivity of the Welfare Quality® broiler chicken protocol to differences between intensively reared indoor flocks: which factors explain overall classification? *Animal* (2017) **11**:244–253. doi:10.1017/S1751731116001476

Butterworth A. EU FP6 Welfare Quality ® Poultry Assessment Systems. *Korean J Poult Sci* (2009) **36**:239～246. doi:10.5536/kjps.2009.36.3.239

Butterworth A. On-farm broiler welfare assessment and associated training. *Rev Bras Cienc Avic* (2013) **15**:71–77. doi:10.1590/S1516-635X2013000200001

Can E, Vieira A, Battini M, Mattiello S, Stilwell G. Consistency over time of animal-based welfare indicators as a further step for developing a welfare assessment monitoring scheme: The case of the Animal Welfare Indicators protocol for dairy goats. *J Dairy Sci* (2017) **100**:9194–9204. doi:10.3168/jds.2017-12825

Can E, Vieira A, Battini M, Mattiello S, Stilwell G. On-farm welfare assessment of dairy goat farms using animal-based indicators: the example of 30 commercial farms in Portugal. *Acta Agric Scand A Anim Sci* (2016) **66**:43–55. doi:10.1080/09064702.2016.1208267

Canali E, Keeling L. Welfare Quality® project: From scientific research to on farm assessment of animal welfare. *Ital J Anim Sci* (2009) **8**:900–903. doi:10.4081/ijas.2009.s2.900

Caplen G, Hothersall B, Nicol CJ, Parker RMA, Waterman-Pearson AE, Weeks CA, Murrell JC. Lameness is consistently better at predicting broiler chicken performance in mobility tests than other broiler characteristics. *Anim Welf* (2014) **23**:179–187. doi:10.7120/09627286.23.2.179

Caroprese M, Casamassima D, Rassu SPG, Napolitano F, Sevi A. Monitoring the on-farm welfare of sheep and goats. *Ital J Anim Sci* (2009)343–354. doi:10.4081/ijas.2009.s1.343

Çavuşoǧlu E, Petek M, Abdourhamane IM, Akkoc A, Topal E. Effects of different floor housing systems on the welfare of fast-growing broilers with an extended fattening period. *Arch Anim Breed* (2018) **61**:9–16. doi:10.5194/aab-61-9-2018

Corazzin M, Piasentier E, Dovier S, Bovolenta S. Effect of summer grazing on welfare of dairy cows reared in mountain tie-stall barns. *Ital J Anim Sci* (2010) **9**: doi:10.4081/ijas.2010.e59

Croyle SL, Nash CGR, Bauman C, LeBlanc SJ, Haley DB, Khosa DK, Kelton DF. Training method for animal-based measures in dairy cattle welfare assessments. *J Dairy Sci* (2018) **101**:9463–9471. doi:10.3168/jds.2018-14469

Czycholl I, Büttner K, Beilage EG, Krieter J. Review of the assessment of animal welfare with special emphasis on the “Welfare Quality® animal welfare assessment protocol for growing pigs.” *Arch Tierzucht* (2015) **58**:237–249. doi:10.5194/aab-58-237-2015

Czycholl I, Grosse Beilage E, Henning C, Krieter J. Reliability of the qualitative behavior assessment as included in the welfare quality assessment protocol for growing pigs. *J Anim Sci* (2017) **95**:3445–3454. doi:10.2527/jas.2017.1525

Dai F, Dalla Costa E, Anne Murray LM, Canali E, Minero M. Welfare conditions of donkeys in Europe: Initial outcomes from on-farm assessment. *Animals* (2016) **6**:8–12. doi:10.3390/ani6010005

Daigle C, Siegford J. Welfare Quality® parameters do not always reflect hen behaviour across the lay cycle in non-cage laying hens. *Anim Welf* (2014) **23**:423–434. doi:10.7120/09627286.23.4.423

Dalla Villa P, Matthews LR, Alessandrini B, Messori S, Migliorati G. Drivers for animal welfare policies in Europe. *OIE Rev Sci Tech* (2014) **33**:39–46. doi:10.20506/rst.33.1.2266

De Jong IC, Hindle VA, Butterworth A, Engel B, Ferrari P, Gunnink H, Perez Moya T, Tuyttens FAM, Van Reenen CG. Simplifying the Welfare Quality® assessment protocol for broiler chicken welfare. *Animal* (2016) **10**:117–127. doi:10.1017/S1751731115001706

De Passillé AM, Rushen J. Food safety and environmental issues in animal welfare. *OIE Rev Sci Tech* (2005) **24**:757–766. doi:10.20506/rst.24.2.1599

De Rosa G, Grasso F, Pacelli C, Napolitano F, Winckler C. The welfare of dairy buffalo. *Ital J Anim Sci* (2009) **8**:103–116. doi:10.4081/ijas.2009.s1.103

De Rosa G, Grasso F, Winckler C, Bilancione A, Pacelli C, Masucci F, Napolitano F. Application of the Welfare Quality protocol to dairy buffalo farms: Prevalence and reliability of selected measures. *J Dairy Sci* (2015) **98**:6886–6896. doi:10.3168/jds.2015-9350

De Rosa G, Napolitano F, Grasso F, Pacelli C, Bordi A. On the development of a monitoring scheme of buffalo welfare at farm level. *Ital J Anim Sci* (2005) **4**:115–125. doi:10.4081/ijas.2005.115

De Vries M, Bokkers EAM, van Schaik G, Engel B, Dijkstra T, de Boer IJM. Improving the time efficiency of identifying dairy herds with poorer welfare in a population. *J Dairy Sci* (2016) **99**:8282–8296. doi:10.3168/jds.2015-9979

De Vries M, Engel B, Den Uijl I, Van Schaik G, T. Dijkstra, De Boer IJM, Bokkers EAM. Assessment time of the Welfare Quality® protocol for dairy cattle. *Anim Welf* (2013) **22**:85–93. doi:10.7120/09627286.22.1.085

Dippel S, Dolezal M, Brenninkmeyer C, Brinkmann J, March S, Knierim U, Winckler C. Risk factors for lameness in cubicle housed Austrian Simmental dairy cows. *Prev Vet Med* (2009) **90**:102–112. doi:10.1016/j.prevetmed.2009.03.014

Dippel S, Leeb C, Bochicchio D, Bonde M, Dietze K, Gunnarsson S, Lindgren K, Sundrum A, Wiberg S, Winckler C, et al. Health and welfare of organic pigs in Europe assessed with animal-based parameters. *Org Agric* (2014) **4**:149–161. doi:10.1007/s13165-013-0041-3

Domenico V, Tamaio JE, Luigi B, Esterina DC. Development of a new approach to evaluate buffalo welfare, the ruminant welfare® project. *Rev Colomb Ciencias Pecu* (2017) **30**:231–233.

Edgar JL, Mullan SM, Pritchard JC, McFarlane UJC, Main DCJ. Towards a “good life” for farm animals: Development of a resource tier framework to achieve positive welfare for laying hens. *Animals* (2013) **3**:584–605. doi:10.3390/ani3030584

Edwards SA. Experimental welfare assessment and on-farm application. *Anim Welf* (2007) **16**:111–115.

EFSA Panel on Animal Health and Welfare. Scientific Opinion on the assessment of dairy cow welfare in small-scale farming systems. *EFSA J* (2015) **13**: doi:10.2903/j.efsa.2015.4137

EFSA Panel on Animal Health and Welfare. Scientific Opinion on the assessment of studies on the use of carbon dioxide for stunning rabbits. *EFSA J* (2015) doi:10.2903/j.efsa.2015.4022

EFSA Panel on Animal Health and Welfare. Slaughter of animals: poultry. *EFSA J* (2019) **17**:e05849.

EFSA Panel on on Animal Health and Welfare. Killing for purposes other than slaughter: poultry. *EFSA J* (2019) doi:10.2903/j.efsa.2019.5850

Endres MI, Lobeck-Luchterhand KM, Espejo LA, Tucker CB. Evaluation of the sample needed to accurately estimate outcome-based measurements of dairy welfare on farm. *J Dairy Sci* (2014) **97**:3523–3530. doi:10.3168/jds.2013-7464

Fàbrega E, Coma J, Tibau J, Manteca X, Velarde A. Evaluation of parameters for monitoring welfare during transport and lairage at the abottoir in pigs. *Anim Welf* (2007) **16**:201–204.

Ferrante V, Lolli S, Ferrari L, Watanabe TTN, Tremolada C, Marchewka J, Estevez I. Differences in prevalence of welfare indicators in male and female Turkey flocks (Meleagris gallopavo). *Poult Sci* (2019) **98**:1568–1574. doi:10.3382/ps/pey534

Gibbons J, Vasseur E, Rushen J, De Passillé AM. A training programme to ensure high repeatability of injury scoring of dairy cows. *Anim Welf* (2012) **21**:379–388. doi:10.7120/09627286.21.3.379

Giersberg MF, Spindler B, Kemper N. Assessment of plumage and integument condition in dual-purpose breeds and conventional layers. *Animals* (2017) **7**:1–15. doi:10.3390/ani7120097

Goossens X, Sobry L, Ödberg F, Tuyttens F, Maes D, De Smet S, Nevens F, Opsomer G, Lommelen F, Geers R. A population-based on-farm evaluation protocol for comparing the welfare of pigs between farms. *Anim Welf* (2008) **17**:35–41.

Gottardo F, Brscic M, Contiero B, Cozzi G, Andrighetto I. Towards the creation of a welfare assessment system in intensive beef cattle farms. *Ital J Anim Sci* (2009) **8**:325–342. doi:10.4081/ijas.2009.s1.325

Grandin T. Auditing animal welfare and making practical improvements in beef-, pork- and sheep-slaughter plants. *Anim Welf* (2012) **21**:29–34. doi:10.7120/096272812X13353700593400

Grandin T. Auditing animal welfare at slaughter plants. *Meat Sci* (2010) **86**:56–65. doi:10.1016/j.meatsci.2010.04.022

Grandin T. Livestock-handling assessments to improve the welfare of cattle, pigs and sheep. *Anim Prod Sci* (2018) **58**:403–407. doi:10.1071/AN16800

Hansson H, Szczensa-Rundberg M, Nielsen C. Which preventive measures against mastitis can increase the technical efficiency of dairy farms? *Animal* (2011) **5**:632–640. doi:10.1017/S1751731110002247

Harley S, Boyle LA, O’Connell NE, More SJ, Teixeira DL, Hanlon A. Docking the value of pigmeat? Prevalence and financial implications of welfare lesions in Irish slaughter pigs. *Anim Welf* (2014) **23**:275–285. doi:10.7120/09627286.23.3.275

Heath C, Lin Y, Mullan S, Browne WJ, Main D. Implementing Welfare Quality® in UK assurance schemes: Evaluating the challenges. *Anim Welf* (2014) **23**:95–107. doi:10.7120/09627286.23.1.095

Hintze S, Smith S, Patt A, Bachmann I, Würbel H. Are eyes a mirror of the soul? What eye wrinkles reveal about a horse’s emotional state. *PLoS One* (2016) **11**:1–15. doi:10.1371/journal.pone.0164017

Hubbard C, Scott K. Do farmers and scientists differ in their understanding and assessment of farm animal welfare? *Anim Welf* (2011) **20**:79–87.

Ingemann R, Sandøe P, Enemark P, Forkman B. Conflicting goals of welfare assessment schemes: A case study. *Anim Welf* (2009) **18**:487–495.

Jacobs L, Delezie E, Duchateau L, Goethals K, Vermeulen D, Buyse J, Tuyttens FAM. Fit for transport? broiler chicken fitness assessment for transportation to slaughter. *Anim Welf* (2017) **26**:335–343. doi:10.7120/09627286.26.3.335

Jung L, Niebuhr K, Hinrichsen LK, Gunnarsson S, Brenninkmeyer C, Bestman M, Heerkens J, Ferrari P, Knierim U. Possible risk factors for keel bone damage in organic laying hens. *Animal* (2019) **13**:2356–2364. doi:10.1017/S175173111900003X

Keeling LJ, Immink V, Hubbard C, Garrod G, Edwards SA, Ingenbleek P. Designing animal welfare policies and monitoring progress. *Anim Welf* (2012) 95–105. doi:10.7120/096272812X13345905673845

Keeling LJ. An analysis of animal-based versus resource-based comments in official animal welfare inspection reports from organic and conventional farms in Sweden. *Anim Welf* (2009) **18**:391–397.

Keeling LJ. Healthy and Happy: Animal Welfare as an Integral Part of Sustainable Agriculture. *AMBIO A J Hum Environ* (2005) 316–319. doi:10.1639/0044-7447(2005)034[0316:hahawa]2.0.co;2

Kerr AJ, Mullan SM, Main DCJ. A new educational resource to improve veterinary students’ animal welfare learning experience. *J Vet Med Educ* (2013) **40**:1–7. doi:10.3138/jvme.0113-006R

Kirchner MK, Schulze Westerath H, Knierim U, Tessitore E, Cozzi G, Winckler C. On-farm animal welfare assessment in beef bulls: Consistency over time of single measures and aggregated Welfare Quality® scores. *Animal* (2014) **8**:461–469. doi:10.1017/S1751731113002267

Kirchner MK, Westerath SH, Knierim U, Tessitore E, Cozzi G, Pfeiffer C, Winckler C. Application of the Welfare Quality® assessment system on European beef bull farms. *Animal* (2014) **8**:827–835. doi:10.1017/S1751731114000366

Kleinhenz MD, Gorden PJ, Burchard M, Ydstie JA, Coetzee JF. Rapid Communication: Use of pressure mat gait analysis in measuring pain following normal parturition in dairy cows. *J Anim Sci* (2019) **97**:846–850. doi:10.1093/jas/sky450

Knierim U, Winckler C. On-farm welfare assessment in cattle: Validity, reliability and feasibility issues and future perspectives with special regard to the Welfare Quality® approach. *Anim Welf* (2009) **18**:451–458.

Leeb C, Henstridge C, Dewhurst K, Bazeley K. Welfare assessment of working donkeys: Assessment of the impact of an animal healthcare project in West Kenya. *Anim Welf* (2003) **12**:689–694.

Leite LO, Stamm F de O, Garcia R de CM. Indicators to assess goat welfare on-farm in the semiarid region of Brazilian Northeast. *Ciência Rural* (2017) **47**:1–8. doi:10.1590/0103-8478cr20161073

Lin YC, Mullan S, Main DCJ. Use of welfare outcome information in three types of dairy farm inspection reports. *Asian-Australasian J Anim Sci* (2018) **31**:1525–1534. doi:10.5713/ajas.17.0851

Louton H, Erhard M, Wöhr AC. Acquisition of animal-based welfare measures at slaughter of poultry. *Fleischwirtschaft* (2018) **98**:94–98.

Louton H, Keppler C, Erhard M, van Tuijl O, Bachmeier J, Damme K, Reese S, Rauch E. Animal-based welfare indicators of 4 slow-growing broiler genotypes for the approval in an animal welfare label program. *Poult Sci* (2019) doi:10.3382/ps/pez023

Main DCJ, Mullan S. Economic, education, encouragement and enforcement influences within farm assurance schemes. *Anim Welf* (2012) **21**:107–111. doi:10.7120/096272812X13345905673881

Main DCJ, Whay HR, Leeb C, Webster AJF. Formal animal-based welfare assessment in UK certification schemes. *Anim Welf* (2007) **16**:233–236.

Main DCJ. Application of welfare assessment to commercial livestock production. *J Appl Anim Welf Sci* (2009) **12**:97–104. doi:10.1080/10888700902719658

Mattiello S, Battini M, De Rosa G, Napolitano F, Dwyer C. How can we assess positive welfare in ruminants? *Animals* (2019) **9**:758. doi:10.3390/ani9100758

Mattiello S, Klotz C, Baroli D, Minero M, Ferrante V, Canali E. Welfare problems in alpine dairy cattle farms in Alto Adige (Eastern Italian Alps). *Ital J Anim Sci* (2009) 628–630. doi:10.4081/ijas.2009.s2.628

Metz JHM, Dijkstra T, Franken P, Frankena K. Development and application of a protocol to evaluate herd welfare in Dutch dairy farms. *Livest Sci* (2015) **180**:183–193. doi:10.1016/j.livsci.2015.07.002

Meyer MM, Johnson AK, Bobeck EA. A novel environmental enrichment device improved broiler performance without sacrificing bird physiological or environmental quality measures. *Poult Sci* (2019) **98**:5247–5256. doi:10.3382/ps/pez417

Minero M, Dalla Costa E, Dai F, Murray LAM, Canali E, Wemelsfelder F. Use of Qualitative Behaviour Assessment as an indicator of welfare in donkeys. *Appl Anim Behav Sci* (2016) **174**:147–153. doi:10.1016/j.applanim.2015.10.010

Mollenhorst H, Rodenburg TB, Bokkers EAM, Koene P, De Boer IJM. On-farm assessment of laying hen welfare: A comparison of one environment-based and two animal-based methods. *Appl Anim Behav Sci* (2005) **90**:277–291. doi:10.1016/j.applanim.2004.08.011

Møller SH, Hansen SW, Sørensen JT. Assessing animal welfare in a strictly synchronous production system: The mink case. *Anim Welf* (2003) **12**:699–703.

Mondragón-Ancelmo J, García Hernández P, Rojo Rubio R, Arturo Domínguez Vara I, del Campo Gigena M, Napolitano F. Small Flocks Show Higher Levels of Welfare in Mexican Semi-Intensive Sheep Farming Systems. *J Appl Anim Welf Sci* (2020) **23**:348–355. doi:10.1080/10888705.2019.1618303

Mononen J, Møller SH, Hansen SW, Hovland AL, Koistinen T, Lidfors L, Malmkvist J, Vinke CM, Ahola L. The development of on-farm welfare assessment protocols for foxes and mink: The WelFur project. *Anim Welf* (2012) **21**:363–371. doi:10.7120/09627286.21.3.363

Mullan S, Edwards SA, Butterworth A, Ward M, Whay HR, Main DCJ. Welfare science into practice: A successful case example of working with industry. *Anim Welf* (2011) **20**:597–601.

Mullan S, Edwards SA, Butterworth A, Whay HR, Main DCJ. A pilot investigation of possible positive system descriptors in finishing pigs. *Anim Welf* (2011) **20**:439–449.

Mullan S, Edwards SA, Butterworth A, Whay HR, Main DCJ. Inter-observer reliability testing of pig welfare outcome measures proposed for inclusion within farm assurance schemes. *Vet J* (2011) **190**:100–109. doi:10.1016/j.tvjl.2011.01.012

Mullan S, Edwards SA, Butterworth A, Whay HR, Main DCJ. A pilot investigation of Farm Assurance assessors’ attitude to farm animal welfare as a confounding factor to training in pig welfare outcome measures. *Anim Welf* (2011) **20**:413–421.

Mülleder C, Troxler J, Laaha G, Waiblinger S. Can environmental variables replace some animal-based parameters in welfare assessment of dairy cows? *Anim Welf* (2007) **16**:153–156.

Munoz C, Campbell A, Barber S, Hemsworth P, Doyle R. Using longitudinal assessment on extensively managed ewes to quantify welfare compromise and risks. *Animals* (2018) doi:10.3390/ani8010008

Munoz C, Campbell A, Hemsworth P, Doyle R. Animal-based measures to assess the welfare of extensively managed ewes. *Animals* (2018) **8**:1–16. doi:10.3390/ani8010002

Munoz CA, Campbell AJD, Hemsworth PH, Doyle RE. Evaluating the welfare of extensively managed sheep. *PLoS One* (2019) doi:10.1371/journal.pone.0218603

Muri K, Leine N, Valle PS. Welfare effects of a disease eradication programme for dairy goats. *Animal* (2015) **10**:333–341. doi:10.1017/S1751731115000762

Muri K, Stubsjøen SM, Valle PS. Development and testing of an on-farm welfare assessment protocol for dairy goats. *Anim Welf* (2013) **22**:385–400. doi:10.7120/09627286.22.3.385

Napolitano F, De Rosa G, Ferrante V, Grasso F, Braghieri A. Monitoring the welfare of sheep in organic and conventional farms using an ANI 35 L derived method. *Small Rumin Res* (2009) **83**:49–57. doi:10.1016/j.smallrumres.2009.04.001

Napolitano F, Grasso F, Bordi A, Tripaldi C, Saltalamacchia F, Pacelli C, De Rosa G. On-farm welfare assessment in dairy cattle and buffaloes: Evaluation of some animal-based parameters. *Ital J Anim Sci* (2005) **4**:223–231. doi:10.4081/ijas.2005.223

O’Connor CE, Bayvel ACD. Challenges to implementing animal welfare standards in New Zealand. *Anim Welf* (2012) **21**:397–401. doi:10.7120/09627286.21.3.397

Otten ND, Nielsen LR, Thomsen PT, Houe H. Register-based predictors of violations of animal welfare legislation in dairy herds. *Animal* (2014) **8**:1963–1970. doi:10.1017/S1751731114001918

Pairis-Garcia M, Moeller SJ. Animal behavior and well-being symposium: The common swine industry audit: Future steps to assure positive on-farm animal welfare utilizing validated, repeatable and feasible animal-based measures. *J Anim Sci* (2017) **95**:1372–1381. doi:10.2527/jas2016.0960

Pandolfi F, Stoddart K, Wainwright N, Kyriazakis I, Edwards SA. The “Real Welfare” scheme: Benchmarking welfare outcomes for commercially farmed pigs. *Animal* (2017) **11**:1816–1824. doi:10.1017/S1751731117000246

Paton MW, Martin PAJ, Fisher AD. Risk assessment principles in evaluation of animal welfare. *Anim Welf* (2013) **22**:277–285. doi:10.7120/09627286.22.2.277

Peden RSE, Camerlink I, Boyle LA, Akaichi F, Turner SP. Farmer perceptions of pig aggression compared to animal-based measures of fight outcome. *Animals* (2019) **9**:22. doi:10.3390/ani9010022

Peli A, Pietra M, Giacometti F, Mazzi A, Scacco G, Serraino A, Scagliarini L. Survey on animal welfare in nine hundred and forty three Italian dairy farms. *Ital J Food Saf* (2016) **5**:50–56. doi:10.4081/ijfs.2016.5832

Pempek JA. Assessment of Feeding Management in National Dairy FARM Programs. *Tri-State Dairy Nutr Conf* (2016) 83–90.

Peng SJL, Chang FC, Sheng-Ting J, Fei ACY. Welfare assessment of flight-restrained captive birds: Effects of inhibition of locomotion. *Thai J Vet Med* (2013) **43**:235–241.

Pfeifer M, Eggemann L, Kransmann J, Schmitt AO, Hessel EF. Inter- and intra-observer reliability of animal welfare indicators for the on-farm self-assessment of fattening pigs. *Animal* (2019) **16**:1712–1720. doi:10.1017/S1751731118003701

Phythian CJ, Cripps PJ, Michalopoulou E, Jones PH, Grove-White D, Clarkson MJ, Winter AC, Stubbings LA, Duncan JS. Reliability of indicators of sheep welfare assessed by a group observation method. *Vet J* (2012) **193**:257–263. doi:10.1016/j.tvjl.2011.12.006

Phythian CJ, Michalopoulou E, Cripps PJ, Duncan JS, Wemelsfelder F. On-farm qualitative behaviour assessment in sheep: Repeated measurements across time, and association with physical indicators of flock health and welfare. *Appl Anim Behav Sci* (2016) **175**:23–31. doi:10.1016/j.applanim.2015.11.013

Phythian CJ, Michalopoulou E, Duncan JS. Assessing the validity of animal-based indicators of sheep health and welfare: Do observers agree? *Agric* (2019) **9**:88. doi:10.3390/agriculture9050088

Phythian CJ, Mullan S, Butterworth A, Lambton S, Ilić J, Burazerović J, Burazerović E, Leach KA. A pilot survey of farm animal welfare in Serbia, a country preparing for EU accession. *Vet Med Sci* (2017) **3**:208–226. doi:10.1002/vms3.72

Phythian CJ, Toft N, Cripps PJ, Michalopoulou E, Winter AC, Jones PH, Grove-White D, Duncan JS. Inter-observer agreement, diagnostic sensitivity and specificity of animal-based indicators of young lamb welfare. *Animal* (2013) **7**:1182–1190. doi:10.1017/S1751731113000487

Popescu S, Borda C, Diugan EA, Niculae M, Stefan R, Sandru CD. The effect of the housing system on the welfare quality of dairy cow. *Ital J Anim Sci* (2014) doi:10.4081/ijas.2014.2940

Popescu S, Borda C, Diugan EA, Spinu M, Groza IS, Sandru CD. Dairy cows welfare quality in tie-stall housing system with or without access to exercise. *Acta Vet Scand* (2013) doi:10.1186/1751-0147-55-43

Popescu S, Borda C, Sandru CD, Stefan R, Lazar E. The welfare assessment of tied dairy cows in 52 small farms in North-Eastern Transylvania using animal-based measurements. *Slov Vet Res* (2010) **47**:77–82.

Popescu S, Lazar EA, Borda C, Niculae M, Sandru CD, Spinu M. Welfare quality of breeding horses under different housing conditions. *Animals* (2019) **9**:81. doi:10.3390/ani9030081

Raspa F, Cavallarin L, McLean AK, Bergero D, Valle E. A review of the appropriate nutritionwelfare criteria of dairy donkeys: Nutritional requirements, farm management requirements and animal-based indicators. *Animals* (2019) **9**:315. doi:10.3390/ani9060315

Roberts F, Lucas A, Johnson S. The use of a single empirical outcome measure to assess welfare in slaughter plants: Between- and within-sector comparisons of the supply base for a major retail multiple. *Anim Welf* (2012) **21**:139–145. doi:10.7120/096272812X13353700594003

Roe E, Buller H, Bull J. The performance of farm animal assessment. *Anim Welf* (2011) **20**:69–78.

Rouha-Mülleder C, Iben C, Wagner E, Laaha G, Troxler J, Waiblinger S. Relative importance of factors influencing the prevalence of lameness in Austrian cubicle loose-housed dairy cows. *Prev Vet Med* (2009) **92**:123–133. doi:10.1016/j.prevetmed.2009.07.008

Rushen J, Butterworth A, Swanson JC. Animal behavior and well-being symposium:Farm animal welfare assurance: Science and application. *J Anim Sci* (2011) **89**:1219–1228. doi:10.2527/jas.2010-3589

Sadiq MB, Ramanoon SZ, Mossadeq WMS, Mansor R, Syed-Hussain SS. Association between lameness and indicators of dairy cow welfare based on locomotion scoring, body and hock condition, leg hygiene and lying behavior. *Animals* (2017) **5**:79. doi:10.3390/ani7110079

Samuel EK, Whay HR, Mullan S. A preliminary study investigating the physical welfare and welfare code compliance for tethered and free-ranging horses on common land in South Wales. *Anim Welf* (2012) **21**:593–598. doi:10.7120/09627286.21.4.593

Sandgren CH, Lindberg A, Keeling LJ. Using a national dairy database to identify herds with poor welfare. *Anim Welf* (2009) **18**:523–532.

Schanz L, Krueger K, Hintze S. Sex and age don’t matter, but breed type does-factors influencing eye wrinkle expression in horses. *Front Vet Sci* (2019) **6**:154. doi:10.3389/fvets.2019.00154

Scott K, Binnendijk GP, Edwards SA, Guy JH, Kiezebrink MC, Vermeer HM. Preliminary evaluation of a prototype welfare monitoring system for sows and piglets (Welfare Quality® project). *Anim Welf* (2009) **18**:441–449.

Sevi A. Animal-based measures for welfare assessment. *Ital J Anim Sci* (2009)904–911. doi:10.4081/ijas.2009.s2.904

Sharma A, Phillips CJC. Lameness in sheltered cows and its association with cow and shelter attributes. *Animals* (2019) **9**:360. doi:10.3390/ani9060360

Sharma, Kennedy, Schuetze, Phillips. The Welfare of Cows in Indian Shelters. *Animals* (2019) **9**:172. doi:10.3390/ani9040172

Shimmura T, Bracke MBM, De Mol RM, Hirahara S, Uetake K, Tanaka T. Overall welfare assessment of laying hens: Comparing science-based, environment-based and animal-based assessments. *Anim Sci J* (2011) **82**:150–160. doi:10.1111/j.1740-0929.2010.00834.x

Smith RF, Jones ER, Waterman M. Tesco livestock code of practice. *Cattle Pract* (2012) **20**:146–151.

Sørensen JT, Schrader L. Labelling as a tool for improving animal welfare-The pig case. *Agric* (2019) **9**:123. doi:10.3390/agriculture9060123

Souza APO, Molento CFM. The Contribution of Broiler Chicken Welfare Certification at Farm Level to Enhancing Overall Animal Welfare: The Case of Brazil. *J Agric Environ Ethics* (2015) **28**:1033–1051. doi:10.1007/s10806-015-9576-5

Souza APO, Soriano VS, Schnaider MA, Rucinque DS, Molento CFM. Development and refinement of three animal-based broiler chicken welfare indicators. *Anim Welf* (2018) **27**:263–274. doi:10.7120/09627286.27.3.263

Souza APO, Taconeli CA, Plugge NF, Molento CFM. Broiler chicken meat inspection data in brazil: A first glimpse into an animal welfare approach. *Rev Bras Cienc Avic* (2018) **20**:547–554. doi:10.1590/1806-9061-2017-0706

Sprenger M, Vangestel C, Tuyttens FAM. Measuring thirst in broiler chickens. *Anim Welf* (2009) **18**:553–560.

Stocchi R, Mandolini NA, Marinsalti M, Cammertoni N, Loschi AR, Rea S. Animal welfare evaluation at a slaughterhouse for heavy pigs intended for processing. *Ital J Food Saf* (2014) **3**:1712. doi:10.4081/ijfs.2014.1712

Stubsjøen SM, Hektoen L, Valle PS, Janczak AM, Zanella AJ. Assessment of sheep welfare using on-farm registrations and performance data. *Anim Welf* (2011) **20**:239–251.

Tallo-Parra O, Lopez-Bejar M, Carbajal A, Monclús L, Manteca X, Devant M. Acute ACTH-induced elevations of circulating cortisol do not affect hair cortisol concentrations in calves. *Gen Comp Endocrinol* (2017) **240**:138–142. doi:10.1016/j.ygcen.2016.10.007

Temple D, Courboulay V, Manteca X, Velarde A, Dalmau A. The welfare of growing pigs in five different production systems: Assessment of feeding and housing. *Animal* (2012) **6**:656–67. doi:10.1017/S1751731111001868

Temple D, Dalmau A, Ruiz de la Torre JL, Manteca X, Velarde A. Application of the Welfare Quality® protocol to assess growing pigs kept under intensive conditions in Spain. *J Vet Behav Clin Appl Res* (2011) **6**:138–149. doi:10.1016/j.jveb.2010.10.003

Temple D, Manteca X, Dalmau A, Velarde A. Assessment of test-retest reliability of animal-based measures on growing pig farms. *Livest Sci* (2013) **151**:35–45. doi:10.1016/j.livsci.2012.10.012

Temple D, Courboulay V, Velarde A, Dalmau A, Manteca X. The welfare of growing pigs in five different production systems in France and Spain: Assessment of health. *Anim Welf* (2012) **6**:656–667. doi:10.7120/09627286.21.2.257

Trevisi E, Zecconi A, Cogrossi S, Razzuoli E, Grossi P, Amadori M. Strategies for reduced antibiotic usage in dairy cattle farms. *Res Vet Sci* (2014) **96**:229–233. doi:10.1016/j.rvsc.2014.01.001

Trillo Y, Quintela LA, Barrio M, Becerra JJ, Peña AI, Vigo M, Herradon PG. Benchmarking welfare indicators in 73 free-stall dairy farms in northwestern Spain. *Vet Rec Open* (2017) **4**:1–14. doi:10.1136/vetreco-2016-000178

Tuyttens F, Heyndrickx M, De Boeck M, Moreels A, Van Nuffel A, Van Poucke E, Van Coillie E, Van Dongen S, Lens L. Broiler chicken health, welfare and fluctuating asymmetry in organic versus conventional production systems. *Livest Sci* (2008) **113**:123–132. doi:10.1016/j.livsci.2007.02.019

Upjohn MM, Pfeiffer DU, Verheyen KLP. Helping working Equidae and their owners in developing countries: Monitoring and evaluation of evidence-based interventions. *Vet J* (2014) **199**:210–216. doi:10.1016/j.tvjl.2013.09.065

Ursinus WW, Schepers F, De Mol RM, Bracke MBM, Metz JHM, Groot Koerkamp PWG. COWEL: A decision support system to assess welfare of husbandry systems for dairy cattle. *Anim Welf* (2009) **18**:545–552.

Van Asselt ED, Van Bussel LGJ, Van Horne P, Van Der Voet H, Van Der Heijden GWAM, Van Der Fels-Klerx HJ. Assessing the sustainability of egg production systems in the Netherlands. *Poult Sci* (2015) **94**:1742–1750. doi:10.3382/ps/pev165

Van Dijk L, Elwes S, Main DCJ, Mullan SM, Jamieson J. Farmer perspectives on welfare outcome assessment: Learnings from four farm assurance scheme consultation exercises. *Anim Welf* (2018) **27**:1–11. doi:10.7120/09627286.27.1.001

Van Os JMC, Weary DM, Costa JHC, Hötzel MJ, von Keyserlingk MAG. Sampling strategies for assessing lameness, injuries, and body condition score on dairy farms. *J Dairy Sci* (2019) **102**:8290–8304. doi:10.3168/jds.2018-15134

Van Os JMC, Winckler C, Trieb J, Matarazzo S V., Lehenbauer TW, Champagne JD, Tucker CB. Reliability of sampling strategies for measuring dairy cattle welfare on commercial farms. *J Dairy Sci* (2018) **101**:1495–1504. doi:10.3168/jds.2017-13611

Van Os JMC. Considerations for Cooling Dairy Cows with Water. *Vet Clin North Am - Food Anim Pract* (2019) **35**:157–173. doi:10.1016/j.cvfa.2018.10.009

Vanderhasselt RF, Goethals K, Buijs S, Federici JF, Sans ECO, Molento CFM, Duchateau L, Tuyttens FAM. Performance of an animal-based test of thirst in commercial broiler chicken farms. *Poult Sci* (2014) **93**:1327. doi:10.3382/ps.2013-03720

Vannier P, Michel V, Keeling LJ. Science-based management of livestock welfare in intensive systems: Looking to the future. *OIE Rev Sci Tech* (2014) **33**:153–160. doi:10.20506/rst.33.1.2255

Vasseur E, Gibbons J, Rushen J, Pellerin D, Pajor E, Lefebvre D, de Passillé AM. An assessment tool to help producers improve cow comfort on their farms. *J Dairy Sci* (2015) **98**:698–708. doi:10.3168/jds.2014-8224

Vasseur E, Pellerin D, De Passillé AM, Winckler C, Lensink BJ, Knierim U, Rushen J. Assessing the welfare of dairy calves: Outcome-based measures of calf health versus input-based measures of the use of risky management practices. *Anim Welf* (2012) **21**:77–86. doi:10.7120/096272812799129439

Vasseur E. Animal behavior and well-being symposium: Optimizing outcome measures of welfare in dairy cattle assessment. *J Anim Sci* (2017) **95**:1365–1371. doi:10.2527/jas2016.0880

Végh A, Abonyi-Toth Z, Rafai P. Effect of current intensity and duration on the efectiveness of head-only electrical stunning in pigs under commercial conditions. *Acta Vet Hung* (2017) **65**:13–28. doi:10.1556/004.2017.002

Velarde A, Dalmau A. Animal welfare assessment at slaughter in Europe: Moving from inputs to outputs. *Meat Sci* (2012) **92**:244–251. doi:10.1016/j.meatsci.2012.04.009

Vermeer HM, Hopster H. Operationalizing principle-based standards for animal welfare—indicators for climate problems in pig houses. *Animals* (2018) **8**:1–15. doi:10.3390/ani8040044

Vieira A, Battini M, Can E, Mattiello S, Stilwell G. Inter-observer reliability of animal-based welfare indicators included in the Animal Welfare Indicators welfare assessment protocol for dairy goats. *Animal* (2018) **12**:1942–1949. doi:10.1017/S1751731117003597

Viksten SM, Visser EK, Blokhuis HJ. A comparative study of the application of two horse welfare assessment protocols. *Acta Agric Scand A Anim Sci* (2016) **66**:56–65. doi:10.1080/09064702.2016.1186726

Vinco LJ, Archetti IL, Giacomelli S, Lombardi G. Influence of crate height on the welfare of broilers during transport. *J Vet Behav Clin Appl Res* (2016) **14**:28–33. doi:10.1016/j.jveb.2016.06.006

Visser EK, Neijenhuis F, de Graaf-Roelfsema E, Wesselink HGM, de Boer J, van Wijhe-Kiezebrink MC, Engel B, van Reenen CG. Risk factors associated with health disorders in sport and leisure horses in the Netherlands. *J Anim Sci* (2014) **92**:844–855. doi:10.2527/jas.2013-6692

Waiblinger S, Knierim U, Winckler C. The development of an epidemiologically based On-Farm welfare assessment system for use with dairy cows. *Acta Agric Scand A Anim Sci* (2001) **51**:73–77. doi:10.1080/090647001316923108

Webster AJF. The virtuous bicycle: A delivery vehicle for improved farm animal welfare. *Anim Welf* (2009) **18**:141–147.

Webster J. The assessment and implementation of animal welfare: Theory into practice. *OIE Rev Sci Tech* (2005) **24**:723–734. doi:10.20506/rst.24.2.1602

Wellbrock W, Oosting SJ, Bock BB, Antunović B, Kralik G. Harmonization of welfare standards for the protection of pigs with the EU rules: The case of Croatia. *Ital J Anim Sci* (2009) **8**:21–38. doi:10.4081/ijas.2009.s3.21

Whay HR, Leeb C, Main DCJ, Green LE, Webster AJF. Preliminary assessment of finishing pig welfare using animal-based measurements. *Anim Welf* (2007) **16**:209–211.

Whay HR, Main DCJ, Green LE, Webster AJF. An animal-based welfare assessment of group-housed calves on UK dairy farms. *Anim Welf* (2003) **12**:611–617.

Whay HR, Main DCJ, Green LE, Webster AJF. Animal-based measures for the assessment of welfare state of diary cattle, pigs and laying hens: Consensus of expert opinion. *Anim Welf* (2003) **12**:205–217.

Wigham E, Grist A, Mullan S, Wotton S, Butterworth A. The influence of welfare training on bird welfare and carcass quality in two commercial poultry primary processing plants. *Animals* (2019) **9**:584. doi:10.3390/ani9080584

Winckler C, Brinkmann J, Glatz J. Long-tem consistency of selected animal-related welfare parameters in dairy farms. *Anim Welf* (2007) **16**:197–199.

Winckler C, Capdeville J, Gebresenbet G, Hörning B, Roiha U, Tosi M, Waiblinger S. Selection of parameters for on-farm welfare-assessment protocols in cattle and buffalo. *Anim Welf* (2003) **12**:619–624.

Winder CB, Miltenburg CL, Sargeant JM, LeBlanc SJ, Haley DB, Lissemore KD, Godkin MA, Duffield TF. Effects of local anesthetic or systemic analgesia on pain associated with cautery disbudding in calves: A systematic review and meta-analysis. *J Dairy Sci* (2018) **101**:5411–5427. doi:10.3168/jds.2017-14092

Wirthgen E, Goumon S, Kunze M, Walz C, Spitschak M, Tuchscherer A, Brown J, Höflich C, Faucitano L, Hoeflich A. Effects of transport duration and environmental conditions in winter or summer on the concentrations of insulin-like growth factors and insulin-like growth factor-binding proteins in the plasma of market-weight pigs. *Front Endocrinol (Lausanne)* (2018) **9**:1–10. doi:10.3389/fendo.2018.00036

Wiseman-Orr ML, Scott EM, Nolan AM. Development and testing of a novel instrument to measure health-related quality of life (HRQL) of farmed pigs and promote welfare enhancement (Part 2). *Anim Welf* (2011) **20**:549–558.

Zaffino Heyerhoff JC, LeBlanc SJ, DeVries TJ, Nash CGR, Gibbons J, Orsel K, Barkema HW, Solano L, Rushen J, de Passillé AM, et al. Prevalence of and factors associated with hock, knee, and neck injuries on dairy cows in freestall housing in Canada. *J Dairy Sci* (2014) **97**:173–184. doi:10.3168/jds.2012-6367

Zaludik K, Lugmair A, Baumung R, Troxler J, Niebuhr K. Results of the Animal Needs Index (ANI-35L) compared to animal-based parameters in free-range and organic laying hen flocks in Austria. *Anim Welf* (2007) **16**:217–219.

Zuliani A, Mair M, Kraševec M, Lora I, Brscic M, Cozzi G, Leeb C, Zupan M, Winckler C, Bovolenta S. A survey of selected animal-based measures of dairy cattle welfare in the Eastern Alps: Toward context-based thresholds. *J Dairy Sci* (2018) **101**:1428–1436. doi:10.3168/jds.2017-13257

Zuliani A, Romanzin A, Corazzin M, Salvador S, Abrahantes JC, Bovolenta S. Welfare assessment in traditional mountain dairy farms: Above and beyond resource-based measures. *Anim Welf* (2017) **26**:203–211. doi:10.7120/09627286.26.2.203
